# Postoperative Dissecting Ventricular Septal Hematoma: Recognition and Treatment

**DOI:** 10.5402/2011/534940

**Published:** 2011-04-14

**Authors:** Christopher R. Mart, Aditya K. Kaza

**Affiliations:** Departments of Pediatrics and Surgery, School of Medicine, Primary Children's Medical Center, University of Utah, 100 North Mario Capecchi Drive, Salt Lake City, UT 84113, USA

## Abstract

Dissecting ventricular septal hematoma (DVSH) rarely occurs after repair of a ventricular septal defect (VSD) but can lead to serious complications such as septal rupture, myocardial rupture, cardiogenic shock, heart block, outflow obstruction, cardiac tamponade, abscess transformation, and death. This paper describes the diagnosis and management of acute, severe, left ventricular outflow tract obstruction caused by the development of a DVSH after VSD repair.

## 1. Introduction

Dissecting ventricular septal hematoma (DVSH) after repair of ventricular septal defect (VSD) is a rare [1], potentially life threatening [2, 3] complication initiated by surgical disruption of the coronary microcirculation. The resultant bleeding dissects along a plane beneath the endocardium resulting in a hematoma that bulges out into ventricular cavity. The following is a case report of the diagnosis and management of acute, severe, left ventricular outflow tract (LVOT) obstruction caused by the development of a DVSH after VSD repair.

## 2. Case Presentation

**Figure 1:**
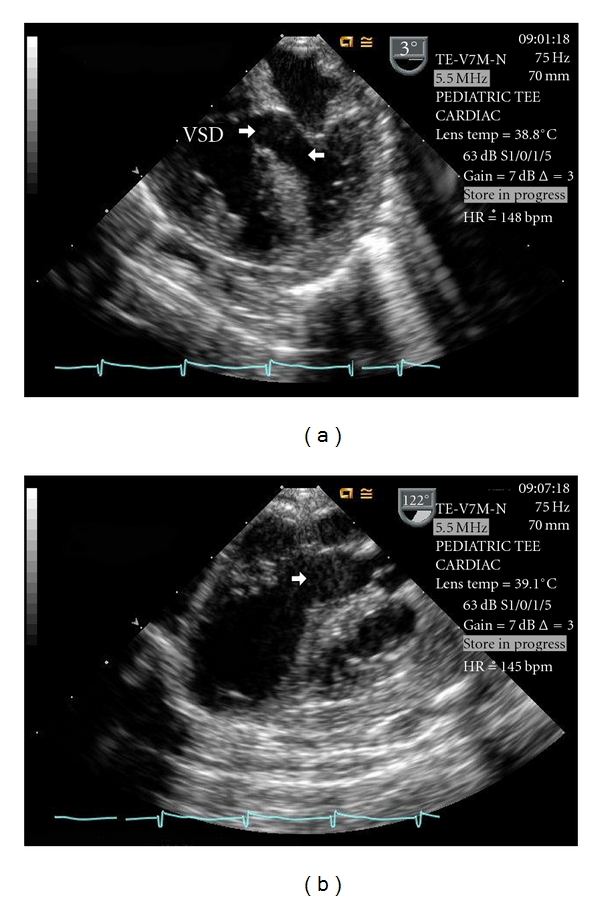
Intraoperative TEE demonstrating a patent LVOT (unlabeled arrow) and the large membranous VSD. Key: LVOT—left ventricular outflow tract, TEE—transesophageal echocardiogram, VSD—ventricular septal defect.

A 7-week-old male infant was noted to be hypoxemic prior to repair of an inguinal hernia. A postoperative echocardiogram demonstrated a large membranous VSD and a long segment coarctation of the aorta. Three months after repair of the coarctation the infant was in congestive heart failure and was brought to the operating room for VSD repair. Preoperative transesophageal echocardiography (TEE) demonstrated a large membranous VSD that extended into the inlet septum and a widely patent left ventricular outflow tract (Figure 1). 

VSD repair was performed using aortobicaval cannulation with mild hypothermia and antegrade cardioplegeic arrest. The echocardiographic findings were confirmed at surgery, and the VSD was closed in the standard manner using a Dacron patch and 5–0 Prolene pledgeted suture. The initial suture line was carried clockwise avoiding the crest of the ventricular septum. Near the septal leaflet of the tricuspid valve, sutures were placed superficially to avoid the conduction system. The VSD was then closed in a counterclockwise fashion staying away from the crest of the ventricular septum, across the ventricular infundibular fold avoiding the aortic annulus, and eventually transitioning to the septal leaflet of the tricuspid valve. The remainder of the VSD underneath the septal leaflet of the tricuspid valve was closed by weaving in and out of the valve leaflet and the VSD patch. The suture was then tied over an autologous pericardial patch. 

**Figure 2:**
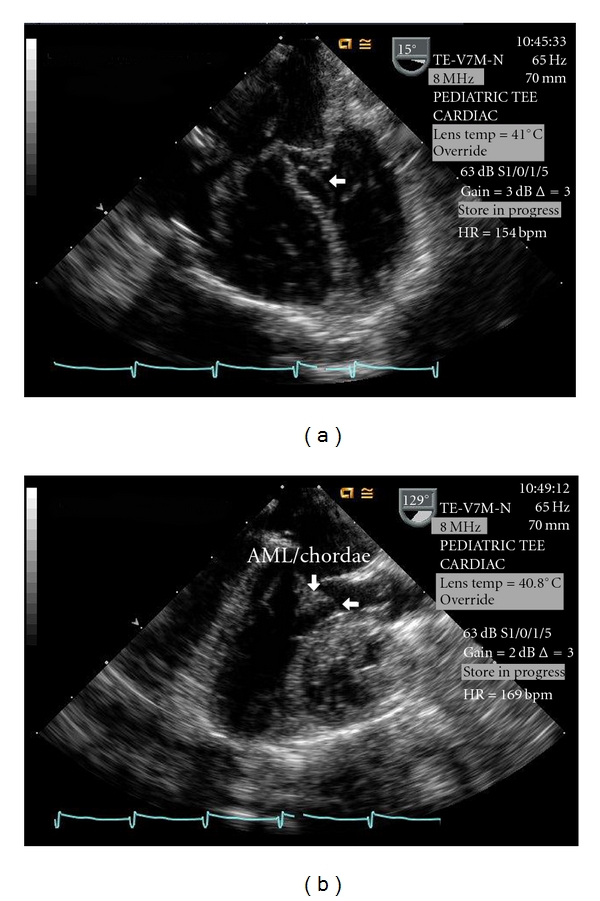
Intraoperative TEE post-VSD repair demonstrating the DVSH (unlabeled arrow) and the systolic narrowing of the LVOT caused by the anterior mitral leaflet/chordal apparatus coming in contact with the DVSH (AML/Chordae). Key: AML/Chordae—anterior mitral leaflet/chordal apparatus, DVSH—dissecting ventricular septal hematoma, LVOT—left ventricular outflow tract, TEE—transesophageal echocardiogram, VSD—ventricular septal defect.

**Figure 3:**
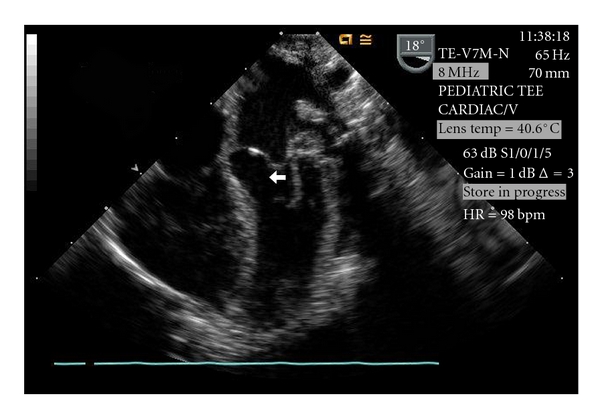
Post-evacuation TEE demonstrating no residual DVSH and a widely patent LVOT. Key: DVSH—dissecting ventricular septal hematoma, LVOT—left ventricular outflow tract, TEE—transesophageal echocardiogram.

Postoperative TEE (Figure 2), performed immediately after coming off of cardiopulmonary bypass, demonstrated an echo lucent region beneath the VSD patch that was surrounded by a thin membrane protruding into the LVOT, findings consistent with a DVSH. During systole the anterior mitral leaflet/chordal apparatus came in contact with the DVSH resulting in severe LVOT obstruction with a peak instantaneous Doppler gradient between 70–80 mmHg which was confirmed by direct pressure measurement. The patient was placed back on cardiopulmonary bypass and an oblique aortotomy was made allowing retraction of the aortic valve leaflets to inspect the ventricular septum. The hematoma was identified and the thin membrane overlying the hematoma was incised and evacuated. The remainder of the hematoma was unroofed and LVOT patency was confirmed with a 9 mm dilator. Postevacuation TEE confirmed a widely patent LVOT with no residual gradient (Figure 3). The LVOT remained widely patent on transthoracic echocardiography performed five days later.

## 3. Discussion

Although rare, DVSH has the potential to cause significant hemodynamic perturbations and may be life threatening. This process, initiated by surgical disruption of the coronary microcirculation, creates a form of myocardial rupture as blood dissects along the spiral planes of the cardiac muscle beneath the endocardium [4]. The resulting hematoma bulges out into the right, left, or both ventricular cavities [4]. This may lead to septal rupture which may create a VSD [5], extension of the hematoma onto the LV free wall with the potential for myocardial rupture [4], development of a communication between the ventricles across their inferior walls without septal rupture [4], cardiogenic shock [2], heart block [6], outflow obstruction [6], cardiac tamponade [6], abscess transformation [7], and death [4]. 

DVSH after VSD repair can be readily diagnosed during intraoperative TEE, and we agree with previous recommendations that intraoperative TEE should be performed in all patients undergoing VSD repair [1]. Both the right and left sides of the ventricular septum must be thoroughly evaluated and the finding of an echo lucent region surrounded by a membrane (the thickness of the membrane varies depending on the level of the hematoma, those deep within the septum have a very thick membrane while those close to the endocardium have a very thin membrane) that protrudes into the cavity of the ventricle (Figure 2) should raise suspicion that there is a septal hematoma present. It must be remembered that since the hematoma is caused by disruption of the coronary microcirculation in a patient that is anticoagulated, small hematomas have the potential to enlarge and cause arrhythmogenic/hemodynamic abnormalities, even after the patient leaves the operating room [1]. For this reason, any suspicious lesion should be thoroughly evaluated and a management strategy determined prior to the patient leaving the operating room. 

Management strategies of DVSH include evacuation [1–3] or observation [5, 8] of the hematoma. Since conservative treatment of DVSH is associated with a mortality rate reported to be as high as 90% [3], we recommend immediate evacuation of the hematoma if the diagnosis is made in the operating room during intraoperative TEE. Because of the potential for reaccumulation of the DVSH if it is evacuated by needle aspiration [1], we recommend that complete unroofing of the hematoma be performed by placing the patient back on cardiopulmonary bypass. 

If the DVSH is diagnosed after the patient has left the operating room, serial echocardiograms should be performed until the size of the DVSH has stabilized and periodically thereafter until the hematoma has either resolved or is noted to be enlarging. If the hematoma enlarges, or causes any other complications, consideration should be given to surgically evacuating the hematoma.
